# The future is precision medicine-guided diagnoses, preventions and treatments for neurodegenerative diseases

**DOI:** 10.3389/fnagi.2023.1128619

**Published:** 2023-03-17

**Authors:** Sharyn L. Rossi, Preeti Subramanian, Diane E. Bovenkamp

**Affiliations:** Scientific Affairs Department, BrightFocus Foundation, Clarksburg, MD, United States

**Keywords:** precision medicine, Alzheimer's disease and related dementias, glaucoma, macular degeneration, biomarkers, artificial intelligence, data sharing, health equity

## Introduction

Precision medicine is essential for the effective prevention and treatment of diseases across diverse populations. Many progressive neurodegenerative diseases, including Alzheimer's disease, age-related macular degeneration (AMD), and glaucoma, involve dysfunction in multiple bodily systems. Nervous, endocrine, immune, cardiac and digestive functions can all contribute to disease progression and the factors that influence each of these systems throughout one's lifetime are exceptionally diverse. It is therefore imperative that we move away from a one-size-fits-all approach to healthcare and lean toward developing more accurate diagnostics and individualized treatment regimen, to better-approach an inclusive healthcare system.

Recent advances in machine-learning algorithms and artificial intelligence (AI) have provided a much-needed framework for data mining and compilation that will lead to the development of the most accurate diagnostics and therapeutic approaches. AI-driven predictive analytics based on neurodegenerative diagnostic measures together with health status (co-morbidities), genetics, environmental exposures, and lifestyle factors would provide an adaptive and harmonized toolbox for healthcare providers to most effectively treat complex, multi-factorial diseases. These algorithms can also mine massive datasets to predict response to treatment, as well as the risk of disease progression. Significant hurdles to overcome include data harmonization and sharing, as well as inclusivity in clinical trials.

Numerous initiatives have been undertaken to bridge inconsistencies in nomenclature and provide easily accessible datasets. Now standard to the field is the NIA-AA Research Framework that biologically defines and clinically stages Alzheimer's disease (Jack et al., 2018). This provided consistent reporting across studies to generate more meaningful information and outcomes. Similar undertakings have occurred in the sub-sectors of reserve and resilience (Stern et al., 2022), astrocyte (Escartin, 2022) and microglial (Paolicelli et al., 2022) nomenclature, and omics taxonomy in Alzheimer's disease (Iturria-Medina et al., 2022), to name a few. Data accessibility has been streamlined through disease data sharing initiatives like the Alzheimer's Disease Neuroimaging Initiative (ADNI) consortium of universities and medical centers in the United States and Canada (Petersen et al., 2010; Alzheimer's Disease Neuroimaging Initiative, 2023), and the National Eye Institute (NEI) Data Commons (NEI Data Commons, 2023), a virtual platform to enable sharing and accessing vision research data and tools for data processing and analysis. While these data repositories are incredibly useful to drive new research findings, a focus on future integration and compilation across resources is essential. A useful model is standardization and harmonization of data collection and protocols across Alzheimer's Disease Research Centers (ADRCs). In a similar data harmonization effort, the NEI, Food and Drug Administration (FDA), and Office of National Coordinator (ONC) for Health Information Technology recently held a joint workshop to promote adopting ocular imaging standards for improving interoperability among ocular imaging modalities and devices to improve biomedical research and patient care (NEI-FDA-ONC Joint Workshop on Promoting Adoption of Ocular Imaging Standards, 2022). These efforts would largely benefit from access to electronic health records as incorporating real world data will result in the best predictive algorithms and treatment paradigms.

## Diagnostics and biomarkers

Identification of novel biomarkers is of upmost importance for early disease detection. While many age-related neurodegenerative diseases are not Mendelian inherited, many genetic risk factors have been identified. Polygenic risk scores (PRSs) derived from genome-wide association studies (GWAS) have shown promise in the stratification of at-risk individuals, across disease stages, and successfully identified novel genes that can be used as new biomarkers and treatment targets. This has enabled early disease management to prevent vision loss and slow cognitive decline. Integrating PRS testing into a clinical setting to screen for neurodegenerative disease is viewed as a cost-effective approach to reduce the economic burden associated with the disease (Hollitt et al., 2022).

For Alzheimer's disease, the most potent risk factor is the *APOEe4* mutation (Raulin et al., 2022), which results in cholesterol dysregulation, but risk varies across ancestral backgrounds and across diseases. There are currently a handful of available diagnostic tests that determine amyloid ratios and *APOE* status. Concomitant studies are looking at other biomarkers including phospho-tau species, neurofilament proteins, and inflammatory markers, to name a few. The ultimate goal should be a multi-analyte panel that distinguishes between multi-etiology dementias, determines disease stage, and predicts treatment efficacy. In-depth algorithms could include vitals, genome sequencing, and longitudinal biomarkers, coupled with neuroimaging scans and neuropsychological tests to provide a wealth of information that will ultimately be needed to build inclusive, comprehensive, and diagnostic algorithms. Without these in-depth diagnostic measures, a precision medicine approach will be lacking. The lack of historically collected data from people with diverse backgrounds, as well as people who are resilient to disease, has extemporaneously thwarted these efforts. To take a holistic approach to medicine, we need to take a holistic approach to data collection.

As neurodegeneration occurs slowly over time, it is essential to advance novel biomarkers that detect diseases at the earliest stages. Clinical trial design can have a profound impact on how we define disease outset (and thus recruitment) and biomarkers can provide personalized targets for future development of therapies. Particularly for Alzheimer's and some vision diseases like AMD and glaucoma, the signs and symptoms are very subtle at the onset, and the symptoms become apparent only at more advanced stages of the disease. Identification of early-stage biomarkers may allow earlier cognition- and/or sight-saving interventions.

## Clinical trial design

Most clinical trials providing evidence for FDA review of an investigative new drug or device do not have a significant (or no) enrollment of non-white participants. Only this year were new guidelines introduced by the FDA to include the enrollment of underrepresented groups into clinical trials (AlzForum News, 2022), and only in 2022 has the Alzheimer's and related dementias field demonstrated the need for more sensitive biomarkers for distinguishing disease differences between races/ethnicities (Kornblith et al., 2022; Schindler et al., 2022; Yeo, 2022). Before we can have an accurate diagnostic test or individualized plan for treatment, we need to address population/country, racial/ethnic, sex-based and other disease differences.

A recent consensus paper discusses other ways to address key recruitment challenges of Alzheimer's disease clinical trials, including strategies to “...encourage broader cognitive screening and earlier AD diagnosis; leverage emerging blood-based biomarker testing, digital technology, and public awareness campaigns; and scale clinical trial architecture in the communities affected by AD” (Langbaum et al., 2022). Women and minorities were only included in clinical trials in a meaningful way in the 1990s in the USA (NIH Revitalization Act of 1993 Public Law 103-43, Subtitle B—Clinical Research Equity Regarding Women and Minorities, 1993), when it was made mandatory by law. Though, even today, many research hypotheses and clinical trials aren't designed properly to focus on understanding the potential biological differences amongst different individuals (and, therefore, the potential for differences in personalized care). We must insist on more directed sex-, racial/ethnic-, socio-economic-, and other-based research and purposeful clinical trial design to detect complex disease differences across populations and provide appropriately tailored treatments.

## Data sharing and diversification

One central aspect to achieving precision medicine is data sharing. There are a significant number of data sharing initiatives in both the neuro and vision space that house invaluable fluid biomarkers, imaging, genetics and other -omics data. Initiatives that seek to standardize data collection across institutions and centers [like what is done to a certain extent by ADRCs (NIA Alzheimer's Disease Research Centers web page, 2021)] would better harmonize these datasets to address demographic, socioeconomic, and sex-based differences in disease incidence, prevalence, and progression. Moreover, the inclusion of electronic health records is a prerequisite for effective precision medicine. The standardization and accessibility of electronic health records across states/provinces and even across countries would provide necessary baseline information on overall health and factors contributing to longevity, risk, and resilience (Blumenthal and James, 2022; Lyketsos et al., 2022). These comprehensive collections of data can then be used to train diagnostic and therapeutic algorithms—resulting in a health score, healthspan trajectory, personalized interventions, and effective treatment plans.

## Look to the eye to lead the way

The retina is an extension of the brain, and neurodegenerative diseases of the eye and brain share common features like cellular senescence, resilience, and vascular components (Rossi et al., 2022a,b). The eye is easily accessible, and the vision research field has led the way in gene therapy to cure a rare inherited retinal degenerative disease (FDA Press Announcements, 2017), retinal imaging to assess dementia (Chan et al., 2017; Cheung et al., 2022; Kwon, 2022), and the first FDA-approved artificial intelligence system for detecting diabetic retinopathy through an eye scan (FDA Press Announcements, 2018). The National Eye Institute's latest strategic plan is leading the way by emphasizing cross-cutting, artificial intelligence, data science, individual quality of life, public health and disparities research, all of which will help clinicians make a go/no go decision on an individual's diagnosis and decide how to pursue personalized care (NEI Strategic Plan: Vision for the Future, 2021).

## Discussion

Precision medicine will play a key role toward a personalized approach needed to treat complex neurodegenerative diseases. This has been steadily gaining traction for some time, but now with the advent of artificial intelligence, new generation genetics and multi-omics screenings (Baxter et al., 2021; Dong et al., 2021; Clark et al., 2022), paired with state-of-the-art technology, the field is approaching a point that personalized medicine could now become a reality. New departments focused on precision medicine are being formed at academic, governmental, non-profit and for-profit institutions around the globe. The most important factor for success will be a collective, collaborative approach—not only across scientific disciplines but also across disease focus.

When developing effective therapies and translating knowledge to clinical care, we must understand how one set of organ systems may respond to treatments directed at another organ system. For instance, some isoforms of risk proteins in the brain (*APOE*) confer resistance in the eye (Klaver et al., 1998; Margeta et al., 2020; Raulin et al., 2022) and any treatment targeting *APOE* would need to take this into account. This issue is further highlighted by recent clinical trials with disease modifying treatments for Alzheimer's disease. It is increasingly clear that the state of the vascular system has a significant role to play when determining efficacy of amyloid immunotherapies. *APOE* status and vascular integrity greatly influence side effects and can make people more or less amenable to specific treatment strategies. These issues demonstrate that cross-fertilization of ideas and research findings/sectors will accelerate the discovery of better diagnostics, preventions, and treatments.

Neurodegenerative diseases require a holistic medical approach. While the field is largely focused on immunotherapies against toxic proteins, we envision a multi-pronged approach that targets multiple facets of disease etiology including protein misfolding, inflammation, and excitotoxicity. Definitive treatments for neurodegenerative diseases of mind and sight will ultimately be personalized therapies, much like the current gold-standards of care for cancer (Ayoub, 2021) and HIV (Pan-American Health Organization and World Health Organization document, 2016). With personalized medicine, an individual will receive the right treatment combination at the right dose at the right time, with continual monitoring and adaptation throughout their lifetime.

## Summary: A roadmap for the future in precision medicine for neurodegenerative diseases

- Biomarkers for identifying high risk individuals and early stages of disease to improve diagnosis and prevention, and to monitor effectiveness of treatments.
- Comply with existing and initiate novel open-source data sharing initiatives, especially for projects involving NIH funding (see mandated data sharing stipulations).
- Ensure scientific rigor and reproducibility, adhering to guidelines set forth by government and other funding entities, including, but not limited to, sex as a biological variable, inclusion of diverse backgrounds and demographics, and publishing negative results to better inform the field.
- Collaborate, discuss, and implement ways to harmonize data, including the involvement of biostatisticians, bioethicists, and artificial intelligence experts across studies, institutes, and neurodegenerative diseases.
- Transform clinical trial initiatives using real world data including coalescing and integrating smaller initiatives and patient cohorts under one umbrella with the goal to uphold health equity.

## Author contributions

DB wrote the initial draft manuscript, with SR and PS equally contributing toward intellectual input, and DB, SR, and PS equally editing toward the final version. All authors read and approved the final manuscript.
